# Relationships between Intensity and Liking for Chemosensory Stimuli in Food Models: A Large-Scale Consumer Segmentation

**DOI:** 10.3390/foods11010005

**Published:** 2021-12-21

**Authors:** Isabella Endrizzi, Danny Cliceri, Leonardo Menghi, Eugenio Aprea, Mathilde Charles, Erminio Monteleone, Caterina Dinnella, Sara Spinelli, Ella Pagliarini, Monica Laureati, Luisa Torri, Alessandra Bendini, Tullia Gallina Toschi, Fiorella Sinesio, Stefano Predieri, Flavia Gasperi

**Affiliations:** 1 Department of Food Quality and Nutrition, Research and Innovation Centre, Fondazione Edmund Mach, Via Edmund Mach 1, 38010 San Michele all’Adige, Italy; leonardo.menghi@fmach.it (L.M.); eugenio.aprea@fmach.it (E.A.); mathildeccharles@gmail.com (M.C.); flavia.gasperi@fmach.it (F.G.); 2 Center Agriculture Food Environment, University of Trento/Fondazione Edmund Mach, Via Edmund Mach 1, 38010 San Michele all’Adige, Italy; danny.cliceri@gmail.com; 3 Department of Technology and Innovation, Center University of Southern Denmark, Campusvej 55, 5230 Odense, Denmark; 4 Department of Agricultural, Food, Environment and Forestry (DAGRI), University of Florence, Via Donizetti 6, 50144 Florence, Italy; erminio.monteleone@unifi.it (E.M.); caterina.dinnella@unifi.it (C.D.); sara.spinelli@unifi.it (S.S.); 5 Department of Food, Environmental and Nutritional Sciences (DeFENS), University of Milan, 20133 Milan, Italy; ella.pagliarini@unimi.it (E.P.); monica.laureati@unimi.it (M.L.); 6 University of Gastronomic Sciences, Piazza Vittorio Emanuele II, 9, 12042 Pollenzo, Italy; l.torri@unisg.it; 7 Department of Agricultural and Food Sciences (DISTAL), Alma Mater Studiorum—University of Bologna, 40126 Bologna, Italy; alessandra.bendini@unibo.it (A.B.); tullia.gallinatoschi@unibo.it (T.G.T.); 8 CREA, Council for Agricultural Research and Economics, Research Center Food & Nutrition, Via Ardeatina 546, 00178 Rome, Italy; fiorella.sinesio@crea.gov.it; 9 Institute for Bioeconomy, CNR, National Research Council, Via Gobetti 101, 40129 Bologna, Italy; stefano.predieri@ibe.cnr.it

**Keywords:** actual liking, perceived intensity, tastes, pungency, consumer segmentation, stated liking

## Abstract

This study, which was conducted as part of the Italian Taste project, was aimed at exploring the relationship between actual liking and sensory perception in four food models. Each food model was spiked with four levels of prototypical tastant (i.e., citric acid, sucrose, sodium chloride, capsaicin) to elicit a target sensation (TS) at an increasing perceived intensity. Participants (N = 2258; 59% women, aged 18–60) provided demographic information, a stated liking for 40 different foods/beverages, and their responsiveness to tastants in water. A food-specific Pearson’s coefficient was calculated individually to estimate the relationship between actual liking and TS responsiveness. Considering the relationship magnitude, consumers were grouped into four food-specific clusters, depending on whether they showed a strong negative (SNC), a weak negative (WNC), a weak positive (WPC), or a strong positive correlation (SPC). Overall, the degree of liking raised in parallel with sweetness responsiveness, fell as sourness and pungency perception increased, and showed an inverted U-shape relationship with saltiness. The SNC clusters generally perceived TSs at higher intensities, except for sourness. Clusters were validated by associating the level of stated liking towards food/beverages; however, some unexpected indications emerged: adding sugar to coffee or preferring spicy foods differentiated those presenting positive correlations from those showing negative correlations. Our findings constitute a step towards a more comprehensive understanding of food preferences.

## 1. Introduction

The pursuit of obtaining nutrition for survival is the most obvious reason for choosing to consume food. However, giving food choices and consumption a mere evolutional connotation is reductive, as widely proved by the amount of evidence suggesting its interdisciplinary and complex nature [1,2]. Although the benefits of having a healthy and balanced dietary style have never been as adequately emphasized than they are now, poor food choices are still a serious threat to our society, as they are at the core of the worldwide spread of many modern non-communicable diseases [3,4]. As a result, other aspects related to the consumers’ food choices have garnered special interest in the last half-century.

It appears axiomatic that we often consume foods for the pleasure imparted by the stimuli intensity (i.e., basic tastes and/or chemesthetic/tactile sensations) they elicit. The assumption that the degree of liking changes as the physical stimulus magnitude changes has been documented for a long time, with the degree of liking rising with the intensity of a sensation until an optimal plateau is reached [5] and then falling again as the sensation continues to increase [6,7,8]. This pattern graphically leads to the now-well-known inverted U-shaped concentration–pleasure curve that was first reported by Joseph Priestley in 1775 and Wilhelm Wurd in 1874 [9], and was later empirically tested [6,7,8,10]. However, a general biphasic curve, whose area under the curve differs as a function of innate taste preferences [11] has previously been proposed [10,12]; the extensive body of literature highlights that sensory-liking patterns vary widely from person to person [5,13,14,15,16,17,18,19,20].

It is worth mentioning that what contributes the most to defining the plateau of the curve is the perceived intensity and not the concentration of a given stimulus [21]. This was previously reported by Moskowitz and colleagues, who added five concentrations of sucrose to different food matrices (i.e., a vanilla pudding, a yellow cake, a cherry-flavoured beverage, and a sucrose solution), and found that optimal liking occurred at a constant degree of sweetness, not at a constant concentration of sucrose [22]. Corroborating these results, Hayes and colleagues observed that liking could be better predicted by using the perceived intensity rather than the stimulus concentration [23,24]. Intuitively, people differing in their sensory responsiveness to tastants will also show different sensory-liking patterns which, in turn, could be used to predict their liking for groups of foods. 

The best-documented genetic source of variation in taste and oral sensation is the perceived bitterness of 6-n-propylthiouracil (PROP) [25,26], which is also deemed to be a proper marker of a heightened response to a wide range of oral stimuli and odour irritants [27,28,29]. Phenotypic responses to PROP allow individuals to be grouped into those who are super sensitive (i.e., supertasters; STs), those who experience moderate responsiveness (i.e., medium tasters; MTs), and those who perceive a compound to be weak or tasteless (i.e., non-tasters; NTs) [30]. PROP Taster Status appears to mediate the shape of individuals’ concentration–pleasure curves for both sweet and salty tastes; however, there is contrasting evidence in this regard, especially for sweet taste. As an example, Looy and Weingarten [31] used hedonic responses to simple sucrose solutions to classify individuals as sweet likers and sweet dislikers. The first group exhibited a rise in liking with increasing concentrations of sucrose, whereas the second group showed a contrasting behaviour. Notably, the authors found a higher percentage of STs to be sweet dislikers and, in parallel, a higher percentage of NTs were sweet likers [31]. Furthermore, sweet dislikers rated the intensity of sucrose as greater than sweet likers, suggesting a possible causal link between the apparent dislike of sweet taste and an enhanced sweet intensity [31]. Other authors [23,32,33], but not all [20,34,35,36], confirmed these results. Nevertheless, it seems that the Sweet Liker Status can reliably predict liking scores for a variety of sweet foods [16] and highly bitter vegetables [20]. In contrast, findings from salty taste are more robust. Hayes and colleagues [15] reported that STs, unlike NTs and MTs, had enhanced responsiveness to saltiness in chicken broth with increasing levels of sodium chloride added, thus resulting in greater changes in liking. This was also true for chips/pretzels and cheeses with commercially available sodium levels, but not for soy sauce, which showed the opposite relationship. Overall, these findings underlie a complex paradigm where individual responsiveness to tastants contributes to the shape of the concentration–pleasure curve, and the latter might be food-specific, at least for salty products [15,37,38].

It is noteworthy that the aforementioned results can also be influenced by biological confounders, such as gender and age, whose effects on oral responsiveness have been widely reported [39,40,41]. As an example, Spinelli and colleagues [20] found a lower percentage of women in the High Sweet Liker group (i.e., those who exhibited a monotonically rising sweetness-liking curve) compared with the Moderate Sweet Liker group (i.e., those whose slopes were less steep than those observed for the Sweet Liker group) [16,20], suggesting that men reach the peak of the concentration–pleasure curve at higher levels of sweetness [20,23]. The same trend was also reported for fatness, with women showing a stronger decline in liking scores than men as the perceived fat intensity in milk/sugar mixtures continued to increase [23]. However, this was not the case for saltiness [15]. This suggests that these findings cannot be extended to all oral sensations.

Age could also affect the plateau of the curve. A plethora of evidence suggests that the detrimental effects of ageing on taste functioning may lead elderly people to like more bitter, salty, and sour tastants than younger adults [42,43,44,45,46], rather than sweet tastants [44,45,46]. In a previous study, Zandstra and De Graaf [46] found that senior adults (65+ yo) were less responsive to the sweetness of sucrose than young adults (19–34 yo), and this led to a leftwards shift in their optimal sucrose peak. Accordingly, senior adults also showed decreased aversion to high citric acid concentrations, which resulted in a less pronounced descending slope for the psychophysical function of citric acid. 

Taken collectively, these findings lead to the conclusion that the causal relationship between perceived intensity and liking is strictly related to the individual and is apparently not generalizable to all oral sensations, other than appearing to be food-specific, at least for salty and sweet foods [21,37,38,47]. Notably, a threefold consideration can be drawn: firstly, the reported methods used to evaluate liking and taste responsiveness differ and are inconsistent with each other. Moreover, examples, where a wide range of sensations were taken into account are relatively rare [29,45,48]. Secondly, it is still remarkably common to assess this relationship using simple aqueous solutions [17,31], although this method is widely recognized to have limited relevance to “real life” perceptions of complex food systems. In this vein, the number of studies using actual food tasting to assess hedonic and intensity responses is increasing [2,15,16,20,23,29,46,48,49,50], but is still limited. Lastly, only a few studies have used a representative sample size [2,16,17,48], which makes it difficult to generalize the abovementioned literature to the whole population.

Hence, the present work, conducted as part of the large-scale Italian Taste project [2], aimed to further explore individual causal relationships between perceived intensity and liking, as measured on real food models modified to elicit different levels of a wide range of target sensations (TS). Moreover, we linked individual variation in sensory-liking patterns with the stated liking for a wide range of food items (selected for having flavour similarity to food model ingredients or to modulated TSs) to discuss its putative influence on food preferences and to hopefully support and promote healthier eating behaviours.

## 2. Materials and Methods 

### 2.1. Participants 

In this paper, data on 2258 Italian subjects (59% women, 18–60 years old) were collected from the 19 laboratory partners of the Italian Taste project over a two-year period (2015–2016) using the procedure and recruiting details reported by Monteleone et al. [2]. Participants had a mean age of 37.8 years (SD: 13.0) and were distributed within the following age and BMI classes: 18–30 (38.0%); 31–45 (28.6%); 46–60 (33.4%); underweight (4%); normal weight (65%); overweight (24%); and 7% obese. 

### 2.2. Products 

#### 2.2.1. Food Models

Four food models were developed using commercially available base ingredients. In the early stages of the Italian Taste project, we developed recipes and preparation procedures to obtain semisolid products that were easy to prepare, preserve, and serve. Pear juice (PJ), chocolate pudding (CP), bean purée (BP), and tomato juice (TJ) were selected as the most appropriate food models to test the responses towards a series of TSs. For each food model, four different concentration levels varying in TS were developed. Each level was expected to increase the perceived TS intensity and, in parallel, increase or decrease the responsiveness to other oral sensations characterizing that specific food model. The ingredient composition and tastant concentration levels within each food model and the oral sensations measured are listed in Table 1. 

The four concentration levels of PJ were prepared to obtain different amounts of citric acid in each sample. The addition of citric acid was expected to increase sourness (TS) while decreasing sweetness. For CP, four concentration levels varying in the amount of sucrose were developed. The addition of sucrose was expected to increase sweetness (TS) while decreasing bitterness and astringency. The four concentration levels of BP were prepared by adding different amounts of sodium chloride. This addition was expected to increase saltiness (TS) and enhance the umami taste. A last series of samples for TJ was prepared to obtain four different capsaicin concentrations. The addition of capsaicin was expected to increase pungency (TS) but also to impact sourness and sweetness. The TS stimuli concentrations were chosen based on published psychophysical data reported by Monteleone and colleagues [2], and verified by preliminary tests with trained panels (unpublished data). 

#### 2.2.2. Aqueous Solutions

To assess individual responsiveness to chemosensory stimulation, seven aqueous solutions containing only one stimulus at a time were included in the study. The stimuli corresponded to the basic tastes (i.e., bitterness, sourness, sweetness, saltiness, and umami), the chemesthetic sensation of pungency, and the tactile sensation of astringency. The aqueous solutions were prepared to elicit an expected moderate/strong sensation on a general Labeled Magnitude Scale (gLMS) presented in vertical position [51]. The concentrations of the chemical reagents (European Pharmacopoeia Reference Standard Sigma-Aldrich, Milano, Italy) were as follows: citric acid, 4 g/kg (sourness); caffeine, 3 g/kg (bitterness); sucrose, 200 g/kg (sweetness); sodium chloride, 15 g/kg (saltiness); monosodium glutamate, 10 g/kg (umami); capsaicin, 1.5 mg/kg (pungent); and aluminium sulphate, 0.8 g/kg (astringency). 

To assess participants’ PROP taster status, supra-threshold 3.2 mM PROP solution was prepared by dissolving 0.5447 g/L of 6-n-propyl-2-thiouracil (European Pharmacopoeia Reference Standard, Sigma-Aldrich, Milano, Italy) in deionized water in accordance with the PROP status assessment procedure described in [52]. 

### 2.3. Evaluation Procedure 

After the recruitment process, respondents were asked to complete an online questionnaire (demographic, socioeconomic, anthropometric, and food habit characteristics) and were invited to attend two sessions in the sensory lab. Liking and perceived intensity responses were collected in two separate sessions over two consecutive days. A hedonic evaluation of the food models (Section 2.3.1), the stated liking questionnaire (Section 2.3.3), and a PROP bitter responsiveness assessment (Section 2.3.2) were completed on day 1, whereas sensory evaluation of aqueous solutions and food models (Section 2.3.2) was performed on day 2. 

All samples were presented in 80 cc plastic cups identified by random three-digit codes. After the evaluation of each sample, participants rinsed their mouths with water, ate unsalted crackers, and then rinsed their mouths with water again for a total of at least 3 min before moving to the next sample. For a detailed overview of the data collection method, see Monteleone et al. [2]. Evaluations were performed in individual booths under white light. Data were collected with Fizz software (ver. 2.51. A86, Biosystèmes, Couternon, France). 

#### 2.3.1. Liking of Food Products

Before starting the hedonic evaluation of food samples, participants were introduced to the Labelled Affective Magnitude scale, presented in vertical position (LAM; [53,54], and were familiarised with it. The scale anchors were spaced according to the values proposed by Cardello and Schutz [54], ranging from ‘greatest imaginable dislike’ (0) to ‘greatest imaginable like’ (100), with ‘neither liked nor disliked’ set at 50. 

The food model series were presented in independent sets, each consisting of four concentration levels (each containing 15 g of product) of the same food model and assessed in a balanced random order. The presentation order of the food models was always the same to avoid carryover effects. PJ was presented as the first set, followed by CP after a ten min break. After a 15 min break, subjects were presented with the BP set, followed by TJ after a ten min break. Subjects were instructed to hold the whole PJ sample in their mouth (or take a full teaspoon of CP, BP, and TJ), wait for 10 s, swallow, and then express their liking.

#### 2.3.2. Intensity Ratings

The intensity of each sensation was rated on a vertical gLMS [51] from ‘not detectable’ (0) to ‘the strongest imaginable sensation of any kind’ (100). Before the first use, extensive training with the scale was performed, as described in detail by Pagliarini et al. [50]. The presentation of food models followed the same method as that used for the liking evaluation. In the latter case, subjects were instructed to evaluate the intensity of the sensations, which varied as a function of the product assessed (Table 1). The overall flavour was also assessed for all products. The order of attribute evaluation was randomized for the tastes and other sensations, whereas the overall flavour was always evaluated last.

To assess the supra-threshold of the key chemosensory stimuli, subjects were also asked to evaluate seven water solutions (10 mL) representing the five basic tastes and astringency presented in random order while capsaicin solution was always evaluated last to avoid carryover effects. During tasting, subjects were instructed to hold the whole water solution sample in their mouth for 3 s, expectorate, wait for 3 s (5 s in the case of bitterness, umami, astringency, and pungency), and rate the intensity of the relevant TS.

To assess the PROP-taster status, subjects were presented with two samples (10 mL) coded with random 3-digit codes and instructed to hold each sample in their mouth for 10 s, expectorate, and then wait for 20 s before evaluating the intensity of the bitterness sensation. For each subject, the average bitterness score across the two samples was used [15,55]. 

#### 2.3.3. Food Preference Questionnaire

Using the IT-Food Preference Questionnaire [2], subjects evaluated the stated liking of 184 food and beverage items grouped into seven categories on a 9-point hedonic scale [56] with the addition of the option ‘never tasted’. Within each product category, the item presentation order and category order were randomized across participants. In the present work, a set of 40 items (fruits and vegetables (*n* = 11), cereals (*n* = 7), cured meat (*n* = 2), dairy (*n* = 4), condiments (*n* = 6), sweets (*n* = 6), hot drinks (*n* = 4)) was considered the most relevant, according to the TSs modulated in the food models and based on food model ingredients 

### 2.4. Data Analyses 

#### 2.4.1. Estimation of the Concentration Level Effect 

For the overall sample of subjects, and within each food model (PJ, BP, CP and TJ), the effect of the concentration level (c1–c4) on liking and perceived intensity was explored by separate one-way analyses of variance (ANOVAs), considering concentration level as a fixed factor. For significant ANOVA effects (*p* < 0.05), the post hoc HSD Tukey’s test for multiple comparisons was used. Additionally, Bonferroni adjustment for multiple testing was applied to account for the high number of estimated models (18). Pearson’s correlation coefficient was then calculated across concentration levels for each product to measure the relationship between liking and perceived intensity for all sensations evaluated.

#### 2.4.2. Cluster Identification

Literature suggests that the relationship between liking and perceived intensity is closely linked to the individual, supposedly not generalizable to all sensations, and product-specific [21,37,38,47]. Hence, to identify segments of subjects with different sensory-liking patterns within each food model, Pearson correlations between individual liking and individual TS intensity perception across the four concentration levels were chosen as segmentation variables. This method allowed the identification of clusters based on individual relationships between liking and perceived intensity and not on the interpretation (visually or statistically) of just hedonic responses, as is commonly proposed for the classification of sweet taste phenotypes [18]. According to the strength (based on Evans’s classification [57]) and direction (positive values denote positive linear correlation and vice versa) of this relationship, consumers were classified into four groups that showed strong negative (SNC: −1 ≤ r < −0.5), weak negative (WNC: −0.5 ≤ r < 0), weak positive (WPC: 0 ≤ r < 0.5), and strong positive (SPC: 0.5 ≤ r ≤ 1) correlations. 

The number of valid data points differed slightly in terms of liking and TS perception intensity assessment from one food model to another due to missing data or the absence of variability. However, the number of missing data points was generally less than 25 per food model.

#### 2.4.3. Cluster Effect Estimation

The four clusters were characterized within each food model. Using separate one-way ANOVAs, we estimated the effect of the clusters (i.e., SNC, WNC, WPC and SPC) on liking scores and the perceived TS intensity at each concentration level, on responsiveness to the seven water solutions, on stated liking scores for food products, and on age. Differences in gender proportion between clusters were tested using the chi-squared test. If a significant effect (*p* < 0.05) emerged from the ANOVA models, Welch’s *t*-test for unequal variances and sample sizes was applied as a post hoc test, thus correcting for the family-wise error-rate. The aforementioned statistical tests (i.e., F, chi-squared, and Welch’s tests) were carried out using a randomization test. This method is free from the constraints of random sampling (of a known error distribution) and equal variances, since these conditions are unlikely to be satisfied in subgroups that are very different in size, as was the case in our study. Accordingly, this non-parametric test is a valid alternative to correctly determine statistical significance by data permutation [58,59]. A particular advantage was gained by using this method, because unbalanced designs and missing values could be easily accommodated [60]. Furthermore, in each test, we performed 10,000 draws to approximate the exact *p*-value with sufficient accuracy. 

Statistical analyses were performed with the R, v. 3.2.3 (RStudio, Inc., Boston, MA, 2015) and the STATISTICA v. 13.1 software (Dell Inc., Tulsa, OK, USA, 2016).

## 3. Results

### 3.1. Perceived Intensity and Liking: Responses from the Whole Panel

#### 3.1.1. Perceived Intensity of Food Models 

**Figure 1:**
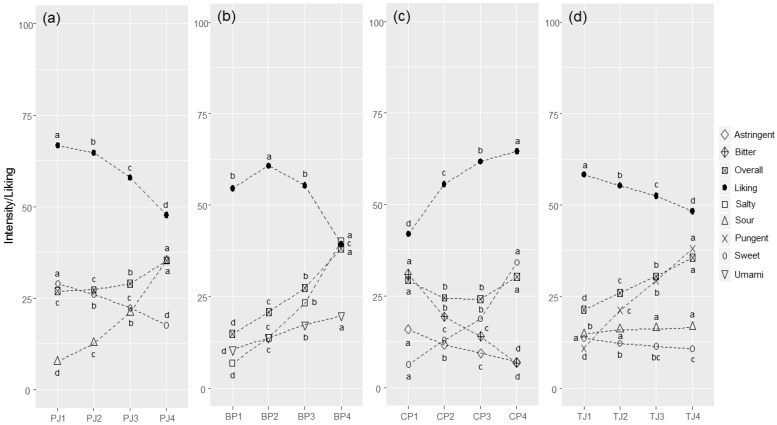
Average perceived intensity (gLM scale) and liking (LAM scale) scores for each food product (PJ = pear juice (**a**); BP = bean purée (**b**); CP = chocolate pudding (**c**); TJ = tomato juice in panel (**d**)) and for each concentration level (1–4), evaluated by the whole panel (PJ: *n* = 2255, BP: *n* = 2256; CP: *n* = 2251; TJ: *n* = 2250). Within each food product, different letters indicate significant differences in intensity/liking between concentration levels (*p* < 0.05).

Overall, the four food models and their four respective concentration levels were found to be comparable in terms of the perceived target sensation (TS) intensity range. Within each food model, the ANOVA model computed on intensity scores showed a significant concentration effect for all attributes (Figure 1a–d). Hence, all concentration levels were significantly different in terms of the perceived TS intensity. 

In Pear juice (PJ, Figure 1a), the perceived sourness intensity increased from ‘weak’ (PJ1, M = 7.7, SD = 9.7) to ‘strong’ (PJ4, M = 35.3, SD = 19.3) (F = 1529.0, *p* < 0.001), the sweetness intensity decreased from ‘strong’ (PJ1, M = 29.0, SD = 15.3) to ‘moderate’ (PJ4, M = 17.7, SD = 14.9) (F = 246.8, *p* < 0.001), and the overall flavour increased from ‘moderate’ (PJ1, M = 26.9, SD = 13.5) to ‘strong’ (PJ4, M = 35.3, SD = 16.8) (F = 164.8, *p* < 0.001). Almost no relationship was found between sweetness and sourness (r = −0.094, *p* < 0.001), whereas the overall flavour was driven by both the perceived sourness (r = 0.483, *p* < 0.001) and the sweetness (r = 0.444, *p* < 0.001).

Bean purée (BP) samples (Figure 1b) showed a constant increase in the perceived saltiness intensity, ranging from ‘weak’ (BP1, M = 6.9, SD = 7.2) to ‘strong’ (BP4, M = 40.1, SD = 18.4) (F = 2742.0, *p* < 0.001). The umami attribute increased from ‘weak’ (BP1, M = 10.5, SD = 11.2) to ‘moderate’ (BP4, M = 19.8, SD = 16.3) (F = 216.1, *p* < 0.001), whereas the overall flavour increased from ‘weak’ (BP1, M = 14.7, SD = 9.7) to ‘strong’ (BP4, M = 38.1, SD = 16.8) (F = 1384, *p* < 0.001). A relationship between salty and umami (r = 0.447, *p* < 0.001) was observed, whereas the overall flavour was driven by both the perceived saltiness (r = 0.791, *p* < 0.001) and umami (r = 0.535, *p* < 0.001).

For the chocolate pudding (CP, Figure 1c), the samples showed an increase in sweetness from ‘weak’ (CP1, M = 6.3, SD = 7.6) to ‘moderate’ (CP4, M = 34.2, SD = 17.3) (F = 2098.0, *p* < 0.001), a decrease in bitterness from ‘moderate’ (CP1 M = 30.9, SD = 17.7) to ‘weak’ (CP4, M = 6.9, SD = 8.8) (F = 1321.0, *p* < 0.001), and a decrease in astringency from ‘moderate’ (CP1, M = 15.9, SD = 15.2) to ‘weak’ (CP4, M = 6.9, SD = 9.7) (F = 222.1, *p* < 0.001), whereas the overall flavour intensity showed a U-shape progression (F = 114.4, *p* < 0.001). We also observed an inverse relationship between sweetness and bitterness (r = −0.327, *p* < 0.001) and almost no relationship with astringency (r = −0.069, *p* < 0.001). Moreover, bitterness and astringency were positively correlated (r = 0.520, *p* < 0.001). Lastly, the overall flavour was found to be positively driven by bitterness (r = 0.427, *p* < 0.001), sweetness (r = 0.357, *p* < 0.001), and astringency (r = 0.341, *p* < 0.001).

Tomato juice (TJ) samples had different pungency levels, with perceived intensity ranging from ‘weak’ (TJ1, M = 10.9, SD = 11.5) to ‘strong’ (TJ4, M = 38.1, SD = 19.3) (F = 1187.0, *p* < 0.001). The sourness level showed low variability among samples (F = 9.7, *p* < 0.001) with the least sour sample (TJ1; M = 14.7, SD = 11.9) being the only significantly different one among the four samples. Sweetness was generally perceived as having a weak intensity, with a decrease from samples TJ1 (M = 13.7, SD = 10.8) to TJ4 (M = 10.9, SD = 11.1) (F = 29.0, *p* < 0.001). Finally, the overall flavour intensity showed a gradual increase from ‘moderate’ (TJ1, M = 21.4, SD = 11.1) to ‘strong’ (TJ4, M = 35.6, SD = 16.3) (F = 444.4, *p* < 0.001). The overall flavour was mainly driven by the pungent sensation (r = 0.734, *p* < 0.001) but also by sourness (r = 0.437, *p* < 0.001). Accordingly, we found a positive relationship between pungency and sourness (r = 0.307, *p* < 0.001).

#### 3.1.2. Liking of Food Products 

Average liking scores and significant differences among developed samples for each food model are reported in Figure 1a–d. 

Overall, liking was found to be linearly related to sweetness: it increased weakly with perceived sweetness for PJ (r = 0.169, *p* < 0.001) and increased moderately for CP (r = 0.317, *p* < 0.001). On the other hand, the linear relationship between liking and perceived sourness intensity was moderately inverse in PJ (r = −0.349, *p* < 0.001) and almost zero in TJ (r = −0.084, *p* < 0.001). Liking also significantly decreased with an increasing level of bitterness (r = −0.328, *p* < 0.001) in CP and a pungent sensation (r = −0.249, *p* < 0.001) in TJ, whereas an inverted U-shape was shown for saltiness (r = −0.337, *p* < 0.001) and umami (r = −0.102, *p* < 0.001) in BP. 

PJ liking (Figure 1a) decreased on average from ‘like slightly’ (PJ1, M = 66.8, SD = 13.0) to ‘dislike slightly’ (PJ4, M = 47.7, SD = 18.4) (F = 713.0, *p* < 0.001). BP liking (Figure 1b) reached a maximum level in BP2 (M = 60.8, SD = 15.1) and then decreased to BP4 (M = 39.3, SD = 20.2) with an inverted U-shape (F = 660.1, *p* < 0.001). CP liking (Figure 1c) increased from ‘dislike moderately’ (CP1, M = 42.0, SD = 19.4) to ‘like slightly’ (CP4, M = 64.5, SD = 16.1) (F = 808.8, *p* < 0.001). The liking for TJ (Figure 1d) decreased from ‘like slightly’ (TJ1, M = 58.4, SD = 16.8) to ‘dislike slightly’ (TJ4, M = 48.3, SD = 22.5) (F = 106.8, *p* < 0.001). 

### 3.2. Perceived Intensity and Liking: Responses by Consumer Clusters 

#### 3.2.1. Cluster Characterization

Table 2 shows the four clusters characterized by the size number, gender percentage, average age, and supra-threshold average responses towards TS in water solution for each product. 

No effect of age was found for any of the food-product-based clusters. In all food models, except for PJ, clusters showed differences in terms of gender: we found a lower percentage of women in the group of subjects showing a strong positive correlation (SPC) between liking and perceived sweetness for CP and perceived pungency for TJ. Furthermore, higher percentages of women were also found in clusters of subjects showing weak and strong negative correlations between liking and perceived saltiness (WNC and SNC) for BP. 

In the Supplementary Materials (Figures S1 and S2, respectively), differences between clusters in terms of liking and perceived TS intensity among the four samples of each food model are depicted. All results included in the supplementary materials are reported and described in the following paragraphs, with each section dedicated to a food model.

#### 3.2.2. Pear Juice

**Figure 2:**
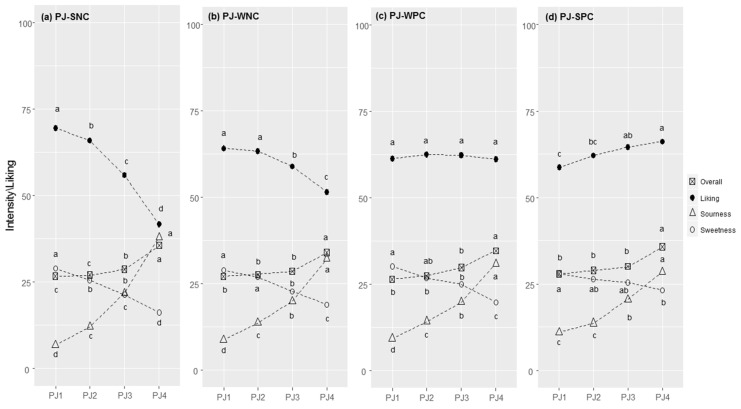
Pear juice (PJ) responses in each cluster (SNC = Strong Negative Correlation (**a**), WNC = Weak Negative Correlation (**b**), WPC = Weak Positive Correlation (**c**), and SPC = Strong Positive Correlation (**d**)): perceived intensity (gLM scale) and liking (LAM scale) averages for each concentration level of citric acid (1–4). Within each cluster, different letters indicate significant differences in intensity/liking between concentration levels (*p* < 0.05).

PJ responses in each cluster are reported in Figure 2a–d. The most populated cluster was the SNC, where liking decreased as perceived sourness increased (N = 1427, 63.7%; r = −0.518, *p* < 0.001). This was followed by the WNC cluster, which showed a similar liking trend but lower differences between samples (N = 391, 17.4%; r = −0.150, *p* < 0.001). In the WPC (weak positive correlation) cluster, samples were equally liked, although the perceived sourness increased (N = 203, 9.1%; r = 0.031, *p* = 0.384), whereas in the SPC cluster, liking increased as perceived sourness increased (N = 219, 9.8%; r = 0.285, *p* < 0.001). 

Liking scores were statistically different between clusters for all concentration levels, with *p*-values always lower than 0.001 (Figure S1a). 

People in the SNC and WNC clusters liked less sour samples (PJ1 and PJ2), whereas PJ3 and PJ4 were liked by those grouped in the WPC and SPC clusters.

Differences in the perceived intensity of sensations among the four samples between clusters were evident, especially for the TS (Figure S2a). For sourness, significant differences between clusters were found for all concentration levels, except for PJ3 (F = 2.1; *p* = 0.097). People in the SNC cluster perceived PJ1 less intensely and PJ4 more intensely than those in the other clusters, whereas the opposite was true for those with SPC. For sweetness, differences between clusters were found for PJ3 (F = 8.1, *p* < 0.001) and PJ4 (F = 17.4, *p* < 0.001), which were perceived as more intense by the SPC compared with the SNC cluster. No significant differences were observed for the overall flavour perception.

Supra-threshold responses toward sensations in water solutions (Table 2) highlighted no significant cluster effects. Significant differences between clusters for the stated level of liking for a series of food ingredients and recipes are reported in Table 3. We found significant differences for 17 out of the 40 foods investigated. The SPC cluster expressed lower liking scores than the other clusters for sweetened tea and coffee, sweet provolone, pear, and lychees but higher ratings for green apples, spicy foods (spicy tomato spaghetti, garlic, olive oil and hot pepper spaghetti, spicy tomato mini pizzas, hot peppers), and unsweetened hot drinks.

#### 3.2.3. Bean Purée

**Figure 3:**
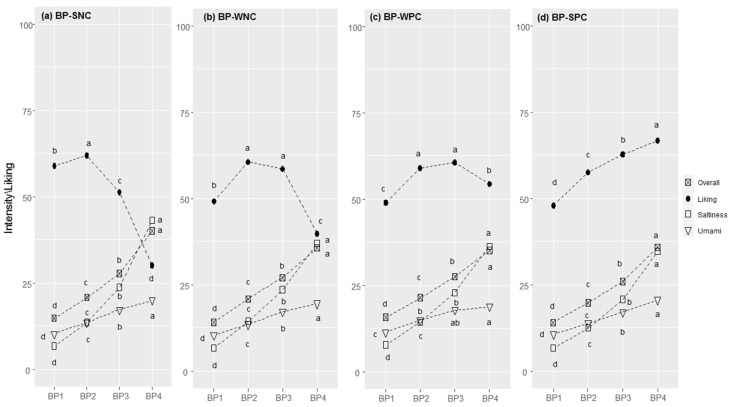
Bean purée (BP) responses in each cluster (SNC = Strong Negative Correlation (**a**), WNC = Weak Negative Correlation (**b**), WPC = Weak Positive Correlation (**c**), and SPC = Strong Positive Correlation (**d**)): perceived intensity (gLM scale) and liking (LAM scale) for bean purée samples at increasing concentrations of sodium chloride (1–4). Within each cluster, different letters indicate significant differences in intensity/liking between concentration levels (*p* < 0.05).

Cluster segmentation highlighted that the correlation between liking and saltiness followed different patterns (Figure 3a–d). Most consumers were in the SNC (N: 1249, 62.3%; r: −0.547, *p* < 0.001), followed by the WNC (N = 488, 21.6%; r = −0.180, *p* < 0.001), SPC (N = 270, 11.9%; r = 0.346, *p* < 0.001), and then the WPC (N = 244, 10.8%; r = 0.024, *p* = 0.453). As the perceived intensity of salty taste increased, three out of the four clusters (i.e., CNS, WNC, WPC) showed inverted U-shaped liking curves with peaks that varied between BP2 and BP3. Conversely, those in the SPC cluster had a level of liking that increased as the perceived saltiness increased.

Liking scores were statistically different between clusters for all concentration levels, showing F-values of more than 8 (*p* < 0.0001, Figure S1b). People in the SNC cluster liked the sample with the lowest concentration of sodium chloride, whereas the opposite was true for those in the SPC cluster, where BP3 and BP4 were the most-liked samples.

The perceived saltiness rating differed significantly between clusters (Figure S2b) for BP3 (F = 3.4, *p* = 0.017) and BP4 (F = 28.9, *p* < 0.001), with the saltiness perceived to be less intense by people in the SPC cluster than those in other clusters. Furthermore, BP4 was perceived as more intense in the SNC cluster than in other groups in terms of both saltiness and overall flavour (F = 13.9, *p* < 0.001; data not shown). 

Supra-threshold responses toward sensations in water solutions (Table 2) highlighted a significant cluster effect on the perceived saltiness (F = 2.8, *p* = 0.041), with an increase in responsiveness from SPC (M = 35.8, SD = 20.1) to SNC (M = 38.5, SD = 20.2). A further significant cluster effect was found for astringency (F = 3.8, *p* = 0.010), with significantly higher levels of responsiveness in the SPC (M = 19.7, SD = 17.8) and SNC (M = 20.1, SD = 18.5) clusters in comparison with those reported for the WNC and WPC clusters (M = 17.9 and M = 16.7, respectively). 

Regarding the stated level of liking, significant differences between clusters for eight food ingredients or recipes were found (Table 3). Compared with the other two clusters, WPC and SPC showed higher levels of liking for spicy foods (spicy salami and spicy tomato spaghetti, spicy salami, and spicy tomato mini pizzas) and sweetened hot drinks.

#### 3.2.4. Chocolate Pudding

**Figure 4:**
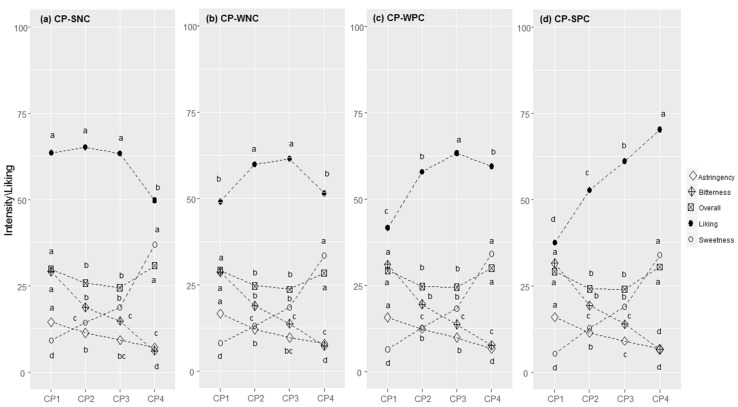
Chocolate Pudding (CP) responses in each cluster (SNC = Strong Negative Correlation (**a**), WNC = Weak Negative Correlation (**b**), WPC = Weak Positive Correlation (**c**), and SPC = Strong Positive Correlation (**d**)): perceived intensity (gLM scale) and liking (LAM scale) for samples at increasing concentrations of sucrose (1–4). Within each cluster, different letters indicate significant differences in intensity/liking between concentration levels (*p* < 0.05).

For CP, the correlation between liking and perceived sweetness followed different patterns in the four clusters (Figure 4a–d). Most consumers were in the SPC cluster (N = 1399, 62.3%) and showed an overall strong positive linear correlation between liking and perceived sweetness (r = 0.522, *p* < 0.001). This was followed, in terms of size, by the WPC (N = 412, 18.3%; r = 0.205, *p* < 0.001), WNC (N = 223, 9.9%; r = −0.130, *p* < 0.001), and SNC (N = 212, 9.4%, r = −0.353, *p* < 0.001) clusters. These clusters showed inverted U-shaped liking curves, with the peak lying between CP2 and CP3, depending on the cluster.

Liking scores were statistically different between clusters for all concentration levels, showing F-values of more than 3.2 (*p* < 0.0199) (Figure S1c). The sweetest sample (CP4) was the most liked in the SPC cluster but the least liked in the SNC cluster. CP1, CP2, and CP3 were equally the most liked in the SNC cluster.

For sweetness, differences between clusters were found for CP1 (F = 21.0, *p* < 0.001), which was perceived as more intense in the SNC and WNC clusters than in other clusters, especially the SPC.

Supra-threshold responses towards sensations in water solution (Table 2) highlighted a cluster effect for the sweet taste intensity (F = 3.5, *p* = 0.017), with an increase in responsiveness for the SPC (M = 39.1, SD = 20.0) to SNC (M = 43.5, SD = 20.3) cluster. No significant differences in intensity between clusters were found for the other sensations in water solutions, except for the PROP responsiveness (F = 3.8, *p* = 0.011), which was perceived to have a higher intensity in those whose level of liking increased with increasing sweetness compared with those presenting a negative correlation. The stated liking scores of 17 food ingredients and recipes differed significantly between clusters (Table 3). Compared with the other clusters, the SPC cluster reported higher liking scores for sweet or sweetened foods (e.g., milk chocolate; sweetened coffee; sweetened hot tea; paprika crisps) and lower liking scores for bitter and pungent products (e.g., unsweetened hot tea and coffee, dark chocolate and dark chocolate pudding, hot pepper, ginger, mustard, and horseradish). For the same foods, SNC showed contrasting behaviours.

#### 3.2.5. Tomato Juice

**Figure 5:**
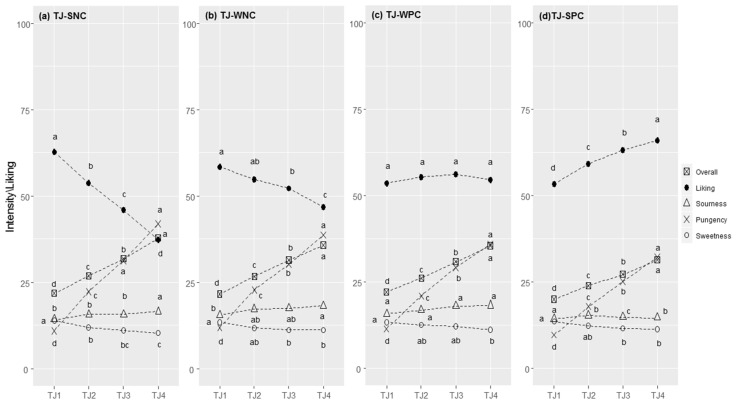
Tomato juice (TJ) responses in each cluster (SNC = Strong Negative Correlation (**a**), WNC = Weak Negative Correlation (**b**), WPC = Weak Positive Correlation (**c**), and SPC = Strong Positive Correlation (**d**)): perceived intensity (gLM scale) and liking (LAM scale) for samples at increasing concentrations of capsaicin (1–4). Within each cluster, different letters indicate significant differences in intensity/liking between concentration levels (*p* < 0.05).

Generally, the correlation between liking and pungency followed an almost linear pattern (Figure 5a–d). Most consumers were in the SNC cluster (N = 980, 43.8%, r = −0.473, *p* < 0.001), followed by the WNC cluster (N = 382, 17.1%; r = −0.222, *p* < 0.001), the WPC cluster (N = 383, 17.1%;), which showed no significant correlation between liking and pungency (r = 0.016, *p* = 0.529), and lastly, the SPC cluster (N = 488, 21.8%; r = 0.239, *p* < 0.001). 

Additionally, here, the liking scores were statistically different between clusters for all concentration levels, with F-values of more than 9.4 (*p* < 0.0001, Figure S1d). The less spicy tomato juice (TJ1) was the most liked for those with a negative correlation between liking and pungency (SNC), whereas subjects in the SPC cluster liked TJ2, TJ3, and TJ4 more than members of the other clusters.

Differences between clusters were found for the pungency of all four samples (*p* < 0.025): people in the SPC cluster perceived all samples as being significantly less intense compared those in the other clusters (Figure S2d). For overall flavour, the same trend was observed (*p* < 0.001). Differences between clusters were found for the sourness of TJ3 and TJ4 (*p* < 0.001), which was perceived to have a weaker intensity in the SPC cluster.

Supra-threshold sensitivity towards sensations in water solution (Table 2) highlighted a cluster effect for pungency intensity (F = 19.4, *p* < 0.001), with a decreased responsiveness from the SNC (M = 49.5, SD = 22.3) to the SPC clusters (M = 40.5, SD = 20.1). The four clusters showed significant differences in stated liking for 14 foods (Table 3). Compared with the other clusters, consumers in the SPC cluster showed a higher liking for spicy foods (e.g., hot pepper; small pizzas with spicy salami; small pizzas with spicy tomato; spaghetti with garlic, olive oil and hot pepper; spicy provolone cheese; spicy salami; spicy tomato) as well as for foods with a stinging taste, such as mustard, horseradish, and ginger. Lastly, the liking for unsweetened hot drinks discriminated between groups: unsweetened coffee was liked by those showing a positive correlation between liking and perceived pungency.

## 4. Discussion

### 4.1. Perceived Intensity and Liking Relationship across the Whole Panel

Overall, the main findings of this study are that the level of liking increased as the perceived intensity of sweetness increased, decreased as the perceived sourness and pungency intensities increased, and showed an inverted U-shape curve for perceived saltiness. The latter result is consistent with prior reports where the hedonic curves for salt in chicken broth and Parmigiano Reggiano cheese had inverted U-shapes [15,61]. Our findings are also consistent with those presented by Zandstra and Graaf [46] for perceived sweetness and sourness in orange beverages, whereas they differ from those presented by other studies [23]. Hayes and Duffy [23], who modelled optimal liking for milk/sugar mixtures, reported that the level of liking increased initially as the sucrose concentration increased, before peaking at 10% sucrose. Furthermore, other authors [62,63], who determined taste perception by presenting subjects with water solutions representing basic tastes at different concentrations, showed that a higher taste intensity rating was associated with a lower level of liking. Here, this was generally true for sourness, saltiness, and pungency, but not for sweetness. Undoubtedly, the works cited differ from each other and from our study in terms of the sample size, methods used, and the number of stimuli and concentration level administered to the subjects. 

Our results consider a response to TS (e.g., sweetness) in the more ecological context of a food model, where other sensory properties (e.g., bitterness) may be inversely correlated and might have affected the results. In pear juice (PJ) and chocolate pudding (CP), inverse relationships between liking and perceived sweetness and sourness were observed. As expected, positive associations between liking and both saltiness and umami were found for bean purée (BP). Sodium chloride and monosodium glutamate, responsible for salt and umami taste perception, respectively, are used as flavour-enhancing agents and are capable of increasing the level of liking for low sodium foods (see among others [64]). As expected, appreciated innate sensations are positively related to liking until an optimum level, which is food-specific, is reached [21], unlike unwelcome sensations, which are negatively correlated.

### 4.2. Stated liking, Actual Liking, and Perceived Intensity in Different Clusters

For CP, a cluster with a distinct aversion to sweet taste was not identified, but rather, groups of subjects that presented inverted U-shape curves with a peak at gradually lower concentrations (moving from strong positive correlation (SPC) to strong negative correlation (SNC) cluster) was found, as previously observed in other studies [16,17,20,36,65]. The SPC cluster was the predominant group gathering for CP, accounting for more than 60% of the subjects, mostly women. This result is consistent with previous reports where the ‘liker’ phenotype accounted for most of the panel involved [7,16,66]. In the least sweet samples (i.e., CP1, CP2), the SNC cluster exhibited a heightened level of responsiveness to sweetness, and this was also confirmed for the sweet water solution. Moreover, we found that those presenting a positive correlation between liking and perceived sweetness (WPC and SPC) had higher levels of supra-threshold responsiveness to the bitterness elicited by PROP. It was reported that those who perceived PROP as the most bitter (PROP super-tasters) had higher levels of sensitivity than non-tasters to various oral stimuli, including other bitter-tasting compounds, acid, salt, sweet substances, and chemical irritants [36,52,67,68]. Furthermore, results by Piochi and colleagues [29] confirmed an overall high level of sensory responsiveness covering different sensory modalities. This could suggest that an increased TS perception has a suppressive function on bitterness perception, causing a consequent increase in liking. 

Tuorila and Pangborn [69] reported the stated level of liking as being a predictor of consumption, and in this sense, our findings for CP are in line with the literature reporting that a greater liking for sweetness is linked to higher consumption of sweet and sweetened foods [17,47,70,71]. People presenting a strong positive correlation between liking and perceived sweetness (SPC) liked sweet or sugary drinks more than those in other clusters. Furthermore, they also rated savoury products, such as paprika crisps, higher than other clusters. The paprika crisps contain sugar as the main ingredient in the paprika seasoning and monosodium glutamate and granulated broth as flavour enhancers (e.g., Pringles Hot Paprika). That partially confirms what was reported by Kim et al. [16]: in addition to sweeter foods, sweet likers preferred savoury foods, particularly those that are meaty and fatty. Beyond that, subjects in the SNC cluster preferred bitter foods, such as dark chocolate, and strong-tasting foods, such as hot-pepper, horseradish, mustard, and ginger.

The clustering obtained by the different hedonic responses to the sour taste in PJ identified a predominant group (more than 60% of the subjects) presenting a negative correlation between liking and perceived sourness (SNC). People in the SPC cluster for PJ, which represented less than 10% of the subjects, were less sensitive to sourness variation than those in the SNC cluster: they perceived the least sour sample (PJ1) as more intense and the sourest one (PJ4) as less intense compared with the other clusters. In this food model, where the perception of sweet and sour varied simultaneously, the intensity perceived may have been affected by individual variations in sensitivity and taste interactions for each subject [72]. Furthermore, PJ3 and PJ4 were perceived as sweeter by those in the SPC cluster, suggesting a higher acuity for sweet taste in subjects with a higher liking for sour foods. The WPC and SPC clusters also showed a greater liking for sour foods, such as green apples, than other groups. This is in accordance with previous studies where a higher liking for sour food items was related to the consumption of sour foods and fruits and vegetables [48,73,74]. Consistent with Törnwall et al. [48], people in the WPC and SPC clusters liked some spicy foods and recipes, together with unsweetened tea and coffee.

As for sweetness, a cluster with a clear aversion to salt taste was not identified. Rather, groups of subjects presented inverted U-shaped curves with a peak at gradually higher concentrations moving from SNC to SPC. This result is in line with the atavistic preference for these two tastes. The predominant group (62.3%) presented an inverted U-shaped distribution with a peak on the second concentration (SNC) and contained a higher percentage of women. This is in line with gender differences found by Hayes et al. [15]. In particular, it was found that women reported a greater liking for broth at sodium levels typically found in commercially available soups. Conversely, the SPC was a small group containing 12% of subjects where liking increased with an increasing perceived saltiness intensity. In samples containing higher concentrations of salt (BP3 and BP4) and for that in water solution, saltiness was perceived at a higher intensity in the SNC cluster than in other clusters, especially the SPC. Furthermore, those presenting a positive correlation between liking and perceived saltiness intensity (WPC and SPC) liked sweetened hot drinks, spicy salami, spaghetti, and pizza with spicy toppings, whereas no association with salty or seasoned foods was found, as previously reported [15,75].

The segmentation carried out through the relationship between liking and perceived pungency identified four groups with homogeneous sizes. The predominant group (44%) was that of those presenting a negative relationship between liking and perceived pungency (SNC). People in this cluster were mostly women who perceived samples at all capsaicin concentrations at a higher intensity than those in the SPC cluster. This is consistent with the results of Spinelli et al. [76], who reported that the choice of spicy options was positively correlated with liking and negatively correlated with the perceived burning intensity and that females were more likely to be chili non-users than males. Alternately, Törnwall et al. [48] reported no differences in the pungency intensity between those who preferred the spicy option to the non-spicy one. In this work, people showing a positive correlation between liking and perceived pungency preferred spicy foods or pungent dressings, such as mustard, horseradish, and ginger, as well as unsweetened coffee.

In each food model, clusters were different from several points of view, but a general rule in terms of cluster size and TS perceived intensity can be seen. For sourness, saltiness and pungency, the SNC cluster was always the biggest group, whereas the SPC cluster was for sweetness. The cluster size gives a general pattern of human perception and liking, showing that the majority of people tends to dislike an increasing perception of saltiness and, conversely, to like sweetness when perceived to a higher extent. This is relevant on the general attempt to decrease salt and sucrose concentrations in the food industry [77]. Considering the failure of many new salt- and sugar-reduced products in the market, our results give also the clue for more effective promoting strategies of healthier products. A holistic consumer-oriented approach, which comprises a detailed education and an attentive study of the complex interplay between lifestyles, hedonics and sensory responsiveness, appears to be the key to pursue a better health-related quality of life for our community.

For saltiness, sweetness, and pungency, those consumers belonging to the SNC cluster perceived the TS at a higher intensity than the other clusters. Despite it being well known that biological variables, such as age, may account for different individual sensory acuity levels, we did not find any evidence of this. Furthermore, the stated level of liking for foods and beverages helped to explain the relationship between actual liking and perceived intensity in the four food-specific clusters.

Puputti et al. [78] showed that taste sensitivities are not related to the stated liking but rather to consumption behaviours (e.g., use frequency) and their tendency to mask or modify tastes (e.g., adding sugar/honey to tea). Here, the stated liking for sweetened coffee was found to be discriminant for the clusters in all food models. Sweetened coffee was preferred by the SNC cluster for PJ and TJ and by the SPC cluster for CP and less preferred by the SNC for BP. This confirmed what was found by Kim et al. [16], where the level of liking for coffee with artificial sweetener discriminated between three groups of high, moderate, and low sweet likers. Additionally, Puputti et al. [78] showed that adding milk to coffee is related to a higher bitter sensitivity, whereas adding sugar or honey to tea is more frequent in those who are less sensitive to sourness. Preferring spicy tomato spaghetti and pizza with spicy toppings discriminated between clusters in at least three food products: in all food models, the SPC cluster showed a higher preference for at least one of these spicy items.

### 4.3. Identification of Taste Liker Phenotypes

In this work, taste and pungency phenotypes were identified according to the strength (based on Evans’s classification [57]) and direction (positive values denote positive linear correlation and vice versa) of the Pearson’s coefficient between the level of liking and the perceived individual TS rating for each product. The use of four defined intervals of the coefficient gave a direct indication of the shapes of the hedonic/intensity relationship curves: two groups presented inverse linear relationships between liking and perceived TS intensity: strong (SNC: −1 ≤ r < −0.5) and weak (WNC: −0.5 ≤ r < 0). The other two groups showed direct weak (WPC: 0 ≤ r < 0.5) and strong (SPC: 0.5 ≤ r ≤ 1) relationships. For the classification of sweet-taste-liker phenotypes, different methodological approaches can be found in the literature. Several studies investigated the hedonic response to sweet taste by describing subgroups of individuals who exhibit a strong liking or aversion by using aqueous sucrose solution-based taste tests. However, the methods used to define these phenotypes vary and are often inconsistent across studies. In a comprehensive review, exploring the strengths and weaknesses of more than seventy papers, Iatridi et al. [18] reported five different classification methods used for sweet-taste-liker phenotype identification. Two are specific to the assessment of hedonic responses to multiple sucrose concentrations. In the first, based on visual discrimination of hedonic response curves, subjects were plotted as a function of concentration [7]. The second, based on statistical discrimination, merged participants into homogenous groups according to their hedonic responses using HCA [16]. The authors of the review did not identify a method that was distinctly superior to others and advocated that a statistically robust and less time-consuming method is needed. Together with a few other papers [19,20], the method proposed here can fill this need, also providing a method for the classification of taste phenotypes other than sweet. Individual Pearson’s coefficients between liking ratings and perceived intensity were also recently used by Spinelli et al. [20] to classify sweet likers using their responses to chocolate pudding samples. They calculated, for each subject, Pearson’s correlation coefficients between liking and all sensations measured and then applied a k-means clustering analysis on r values to identify the segments. They identified three clusters, characterized by specific sensory-liking patterns and optimal levels, which were comparable to those found here for CP (WNC, WPC and SPC). The method proposed here, which just takes TS into account allows the identification of one more cluster: sweet dislikers (SNC). It is important to mention that the shapes of the curves depend on the range of tastant concentrations and the food matrix tested.

### 4.4. Strengths, Weaknesses and Future Perspectives

In this study, the relationship between perceived intensity and hedonics for basic tastes and pungency was investigated using a broad approach and the use of four series of designed food models that varied systematically in the perceived target sensation (TS) intensity from ‘weak’ to ‘strong’ on comparable score ranges. This provided independent measures of liking and intensity performed on the same products. The scaling methods used maximised the validity of the comparison between individuals [79], and the use of a very large ethnically homogeneous adult sample, balanced by gender and age, maximised the ability to associate oral sensory phenotypes with hedonics. However, this advantage probably makes our findings applicable with caution to individuals of diverse ethnicities or cultures, because phenotype identified segments could weigh differently on the total population.

Some of the variables included in this study were directly or indirectly measured using scales, whereas others were self-reported. It should be noted that the self-reported variables could be inaccurate, due to memory and subject bias [80]. Thus, in order to be exhaustive and avoid responses distortion, all the instructions were given both verbally (following a detailed presentation) and submitted on the computer screen during the tasting.

The choice of the method for classifying subjects based on Pearson’s coefficient has the advantage of segmenting according to the direction and linearity of the relationship between liking and TS perceived intensity. In addition, clusters in each food model were very different in size and variability as consequence of data-driven determination. To overcome this drawback, we resorted to permutation methods to define test significance and to approximate exact *p*-value with sufficient accuracy were performed 10000 draws each time. However, this segmentation method does not allow management evaluation cases with zero variance (e.g., people assigning the same score to all the samples): these cases have to be eliminated because it is impossible to calculate the correspondent r coefficient. Despite these limitations, the method used proved to be capable of segmenting consumers according to their individual relationships between liking and perceived TS.

The study could be replicated by adding the data of a further sample of thousand Italian consumers in order to confirm the results obtained here and to obtain a more balanced number of consumers in the various segments of each product. Further research could be dedicated to directly compare the segmentation method proposed here and other different methods, in order to confirm the positive result obtained in this paper.

### 4.5. Conclusions

In conclusion, this study deepened the knowledge on the relationship between the level of liking and the intensity of the perceived sensation and allowed us to identify clusters of consumers based on that relationship in a product context. Findings of this work constitute a further step towards obtaining a more complete understanding of liking for tastes and pungent sensation that takes individual sensory responses into account.

For each food model, the proposed approach allows four clusters with different sensory-hedonic patterns to be identified: for saltiness, sweetness, and pungency, subjects with a strong negative correlation between liking and sensory responsiveness perceived the TS at a higher intensity than other clusters. To make easier study comparisons, a need to standardize method selection for classifying taste phenotypes remains, and the method proposed here could be used for this purpose. Furthermore, large-scale studies should represent a generalizable basis for not only knowledge but also methodology. We also demonstrated that the stated liking towards sweetened coffee can be used to differentiate between TS likers and dislikers, together with some spicy food items. This work points out that the relationship between liking and perceived intensity can predict the level of liking for a variety of food items and can be used to identify groups of consumers with different sensory-liking patterns to develop more effective strategies for understanding food preferences and promoting healthier food behaviours.

## Figures and Tables

**Table 1:** Food models: food product (PJ = Pear Juice, BP = Bean Purée, CP = Chocolate Pudding, and TJ = Tomato Juice), ingredients, ingredient brand, tastant concentration at each level, target, and other measured sensations are reported (tastants responsible for the target sensation are written in bold).

Product	Ingredient (Brand)	Tastant Concentrations (g/kg)				TS	Other Sensations
PJ	**Citric acid solution (Sigma-Aldrich, Italy)**	**5**	**20**	**40**	**80**	**Sourness**	Sweetness
	Pear juice (Santal, Parmalat S.p.A., Italy)						Overall flavour
	Sucrose (Zucchero, Eridania S.p.A, Italy)						
	Water						
BP	**Sodium chloride solution (Sigma-Aldrich, Italy)**	**2.0**	**6.1**	**10.7**	**18.8**	**Saltiness**	Umami
	Purée powder mix (Pfanni, Unilever, Italy)						Overall flavour
	Cannellini beans (Cannellini al vapore, Bonduelle Italy, S.p.A., Italy)						
	Water						
CP	**Sucrose (Zucchero, Eridania S.p.A, Italy)**	**38**	**83**	**119**	**233**	**Sweetness**	Bitterness
	Chocolate pudding mix (Budino da zuccherare, Cameo S.p.A., Italy)						Astringency
	Cocoa powder (Cacao Amaro Perugina, Nestlé, Italy)						Overall flavour
	Water						
TJ	**Capsaicin solution (Sigma-Aldrich, Italy)**	**3 × 10^−4^**	**6.8 × 10^−4^**	**10.1 × 10^−4^**	**15.8 × 10^−4^**	**Pungency**	Sourness
	Peeled tomatoes (Pomodori pelati, Mutti S.p.A., Italy)						Sweetness
	Sodium chloride (Sigma-Aldrich, Italy)						Overall flavour
	Water						

**Table 2:** For each food model, the sample size (PJ = Pear Juice, BP = Bean Purée, CP = Chocolate Pudding, and TJ = Tomato Juice), gender distribution, mean age, and mean perceived target sensation intensity in water are reported for all clusters (SNC = Strong Negative Correlation, WNC = Weak Negative Correlation, WPC = Weak Positive Correlation, and SPC = Strong Positive Correlation). Significant differences are presented in bold (*p* < 0.05). Within each row, different letters indicate significant differences between clusters (*p* < 0.05).

Product	Cluster					*p*-Value
	SNC	WNC	WPC	SPC	NA *	
PJ						
N(%) 2240	1427(63.7)	391(17.4)	203(9.1)	219(9.8)	18	
Gender (Female %)	57.9	59.3	59.1	58.4		0.963
Age (years)	37.4	38.4	38.6	38.5		0.267
Sourness in water	34.2	33.9	33.0	30.9		0.142
BP						
N(%) 2251	1249(62.3)	488(21.6)	244(11.9)	270(10.8)	7	
Gender (Female %)	60.5 ^b^	60.7 ^b^	50.8 ^a^	51.9 ^a^		**0.003**
Age (years)	38.3	37.7	36.5	36.5		0.074
Saltiness in water	38.5 ^b^	36.1 ^a^	36.4 ^ab^	35.8 ^a^		**0.041**
CP						
N(%) 2246	212(9.4)	223(9.9)	412(18.3)	1399(62.3)	12	
Gender (Female %)	63.2 ^b^	63.2 ^b^	65.0 ^b^	55.2 ^a^		**<0.001**
Age (years)	39.6	38.6	37.6	37.4		0.080
Sweetness in water	43.5 ^b^	40.1 ^a^	40.6 ^a^	39.1 ^a^		**0.017**
TJ						
N(%) 2233	980(43.8)	382(17.1)	383(17.1)	488(21.8)	25	
Gender (Female %)	62.7 ^c^	61.5 ^bc^	55.9 ^ab^	50.0 ^a^		**<0.001**
Age (years)	37.4	39.3	37.0	37.7		0.056
Pungency in water	49.5 ^a^	48.0 ^a^	44.6 ^b^	40.5 ^c^		**<0.001**

* NA = number of not classified consumers (Section 2.4.2 for details).

**Table 3:** Average stated liking scores for 40 food products presented in clusters (SNC = Strong Negative Correlation, WNC = Weak Negative Correlation, WPC = Weak Positive Correlation, and SPC = Strong Positive Correlation) for pear juice (PJ), bean purée (BP), chocolate pudding (CP), and tomato juice (TJ). Significant differences within each food model are reported in bold and different letters indicate significant differences in stated liking between clusters (*p* < 0.05).

Food Category	Food Ingredient or Recipe	PJ					BP					CP					TJ				
		SNC	WNC	WPC	SPC	*p*	SNC	WNC	WPC	SPC	*p*	SNC	WNC	WPC	SPC	*p*	SNC	WNC	WPC	SPC	*p*
Fruit and Veg	Beans	7.2	7.1	7.3	7.2	0.583	7.2	7.2	7.2	7	0.587	7.3	7.4	7.2	7.1	0.165	7.2	7.1	7.2	7.2	0.757
	Coconut	6.8	6.8	6.9	6.6	0.363	6.7	6.7	7	6.8	0.157	6.9	6.9	6.6	6.8	0.078	6.8	6.6	6.7	6.8	0.519
	Goji	5.7	5.8	6	5.5	0.280	5.8	5.7	5.4	5.8	0.448	5.9	5.5	5.8	5.7	0.331	5.6	5.7	5.9	5.8	0.148
	Green apples	6.5 ^a^	6.5 ^a^	6.9 ^b^	6.8 ^b^	**0.004**	6.6	6.4	6.6	6.6	0.301	6.6	6.6	6.7	6.6	0.725	6.6	6.6	6.6	6.6	0.926
	Legume soup	7.4	7.3	7.6	7.5	0.350	7.5	7.5	7.3	7.4	0.423	7.8 ^c^	7.7 ^bc^	7.5 ^b^	7.3 ^a^	**<0.001**	7.4	7.4	7.5	7.4	0.551
	Lychees	5.9 ^b^	5.7 ^ab^	6 ^b^	5.3 ^a^	**0.035**	5.9	5.7	5.7	5.7	0.532	6.1	5.8	5.9	5.7	0.280	5.7 ^ab^	5.6 ^a^	6 ^bc^	6.1 ^c^	0.015
	Mango	6.1 ^a^	6.1 ^a^	6.4 ^b^	5.8 ^a^	**0.036**	6.1	6	6.1	5.9	0.545	6.3	6.2	6	6	0.224	6,0	6,0	6,0	6.2	0.592
	Pear	7.2 ^b^	7 ^a^	7.1 ^ab^	6.9 ^a^	**0.005**	7.2 ^b^	7.1 ^ab^	7.1 ^b^	6.9 ^a^	**0.045**	7.1	7.1	7	7.2	0.107	7.2 ^bc^	7.3 ^c^	7 ^ab^	7 ^a^	**0.030**
	Tangerine	7.6	7.6	7.8	7.5	0.221	7.6	7.6	7.4	7.6	0.361	7.6	7.7	7.6	7.6	0.855	7.6	7.6	7.6	7.5	0.703
	Tomatoes	7.8	7.7	8	7.8	0.092	7.8	7.7	7.8	7.8	0.374	7.8	7.8	7.7	7.8	0.925	7.9	7.7	7.8	7.7	0.098
	Yellow apples	7.1	6.9	7	6.8	0.051	7	7.1	7	6.9	0.652	7	7	7	7	0.946	7.1 ^c^	7.1 ^bc^	6.9 ^ab^	6.9 ^a^	**0.011**
Cereals	Garlic, olive oil and hot pepper spaghetti	7 ^a^	7 ^ab^	7.3 ^c^	7.3 ^bc^	**0.049**	7	6.9	7.2	7.2	0.151	7	7.3	6.8	7	0.052	6.5 ^a^	7.1 ^b^	7.5 ^c^	7.6 ^c^	**<0.001**
	Paprika crisps	5.7	5.6	5.8	5.8	0.610	5.6	5.7	5.9	5.8	0.346	5.5 ^a^	5.5 ^a^	5.5 ^a^	5.8 ^b^	**0.025**	5.6	5.6	5.8	5.9	0.138
	Spicy salami mini pizzas	5.7	5.8	5.8	6.1	0.252	5.7 ^a^	5.6 ^a^	6 ^b^	6.2 ^b^	**0.004**	5.6 ^ab^	6 ^c^	5.5 ^a^	5.8 ^bc^	**0.028**	5.3 ^a^	5.8 ^b^	6.1 ^b^	6.5 ^c^	**<0.001**
	Spicy tomato mini pizzas	5.9 ^a^	6.1 ^ab^	6.1 ^ab^	6.3 ^b^	**0.048**	6 ^a^	5.8 ^a^	6.3 ^b^	6.4 ^b^	**0.001**	6.1	6.1	5.8	6.1	0.138	5.5 ^a^	6 ^b^	6.4 ^c^	6.8 ^d^	**<0.001**
	Spicy tomato spaghetti	6.4 ^a^	6.5 ^ab^	6.7 ^ab^	6.8 ^b^	**0.023**	6.5 ^b^	6.2 ^a^	6.6 ^b^	6.7 ^b^	**0.046**	6.6	6.8	6.3	6.4	0.083	5.8 ^a^	6.6 ^b^	6.9 ^c^	7.3 ^d^	**<0.001**
	Tomato bruschetta	7.8	7.8	8.1	7.9	0.073	7.8	7.8	7.7	7.9	0.354	7.7	7.8	7.8	7.8	0.349	7.9	7.8	7.9	7.7	0.244
	Tomato spaghetti	7.6	7.6	7.7	7.5	0.481	7.6	7.5	7.7	7.6	0.368	7.3 ^a^	7.6 ^b^	7.7 ^b^	7.6 ^b^	**0.034**	7.7	7.6	7.6	7.5	0.383
Dairy	Spicy provolone cheese	6.2 ^a^	6.4 ^a^	6.8 ^b^	6.4 ^a^	**0.008**	6.3	6.2	6.4	6.6	0.098	6.6 ^b^	6.7 ^b^	6.1 ^a^	6.3 ^a^	**0.007**	5.9 ^a^	6.4 ^b^	6.6 ^b^	6.9 ^c^	**<0.001**
	Sweet provolone cheese	6.8 ^b^	6.7 ^ab^	7.2 ^c^	6.4 ^a^	**<0.001**	6.7	6.8	6.7	6.8	0.602	6.7	6.9	6.6	6.8	0.213	6.7	6.7	6.8	6.8	0.800
	Vanilla yogurt	6	5.9	5.8	5.8	0.244	5.9	6	6.1	5.9	0.829	5.8	5.7	5.9	6	0.234	6	5.8	6.1	5.9	0.174
	Whole white yogurt	6.2	6.4	6.5	6.3	0.078	6.3	6.1	6.2	6.1	0.092	6.7 ^c^	6.5 ^bc^	6.3 ^ab^	6.1 ^a^	**0.001**	6.2	6.2	6.4	6.2	0.338
Cured meat	Salami	7.2	7.2	7.4	7	0.157	7.2	7.1	7.3	7.4	0.060	7.1	7.2	7.1	7.3	0.131	7.2	7.1	7.2	7.3	0.311
	Spicy salami	6	6	6.3	6.3	0.219	6 ^ab^	5.8 ^a^	6.3 ^bc^	6.4 ^c^	**0.008**	6.1	6.4	5.9	6	0.095	5.5 ^a^	6.1 ^ab^	6.3 ^b^	6.9 ^c^	**<0.001**
Hot drinks	Sweetened coffee	6.1 ^b^	5.8 ^a^	5.9 ^ab^	5.6 ^a^	**0.046**	5.8 ^a^	6.1 ^b^	6.3 ^b^	6.3 ^b^	**0.005**	5.2 ^a^	5.7 ^b^	5.8 ^b^	6.2 ^c^	**<0.001**	6.1 ^b^	6.1 ^b^	5.8 ^a^	5.7 ^a^	**0.015**
	Sweetened hot tea	6.3 ^c^	5.9 ^ab^	6.2 ^bc^	5.8 ^a^	**0.009**	6 ^a^	6.2 ^ab^	6.4 ^b^	6.4 ^b^	**0.043**	5.6 ^a^	5.9 ^a^	5.8 ^a^	6.4 ^b^	**<0.001**	6.2	6.2	6	6	0.189
	Unsweetened coffee	4.7 ^a^	4.8 ^ab^	5.3 ^c^	5.2 ^bc^	**0.007**	4.8	4.9	4.8	4.6	0.649	6.1 ^c^	5.4 ^b^	5.2 ^b^	4.4 ^a^	**<0.001**	4.5 ^a^	4.8 ^a^	5.1 ^b^	5.2 ^b^	**<0.001**
	Unsweetened hot tea	5.1 ^a^	5.3 ^ab^	5.6 ^b^	5.5 ^b^	**0.037**	5.4 ^b^	5.1 ^a^	5.1 ^ab^	5 ^a^	**0.049**	6.1 ^c^	5.6 ^b^	5.6 ^b^	5 ^a^	**<0.001**	5.2	5.2	5.3	5.4	0.539
Condiments	Ginger	5.8	5.8	6.1	5.9	0.212	5.9	5.6	5.8	5.7	0.337	6.3 ^b^	6.1 ^b^	5.8 ^a^	5.7 ^a^	**<0.001**	5.6 ^a^	5.8 ^a^	5.8 ^a^	6.1 ^b^	**0.007**
	Horseradish	4.6 ^a^	4.8 ^a^	5.6 ^b^	4.7 ^a^	**0.004**	4.8	4.8	4.6	4.6	0.829	5.2 ^b^	5 ^b^	4.8 ^ab^	4.6 ^a^	**0.019**	4.5 ^a^	4.5 ^a^	5 ^b^	5.3 ^b^	**<0.001**
	Hot pepper	6 ^a^	6.2 ^ab^	6.5 ^b^	6.5 ^b^	**0.005**	6.1	6	6.3	6.3	0.143	6.3 ^ab^	6.6 ^b^	6.1 ^a^	6.1 ^a^	**0.036**	5.3 ^a^	6.3 ^b^	6.7 ^c^	7.3 ^d^	**<0.001**
	Mustard	4.8	4.8	5.2	4.9	0.247	5	4.6	4.8	4.8	0.136	5.4 ^b^	5.2 ^b^	5.1 ^b^	4.7 ^a^	**<0.001**	4.6 ^a^	4.9 ^ab^	5 ^bc^	5.3 ^c^	**<0.001**
	Soy sauce	5.5	5.6	5.7	5.5	0.803	5.6	5.5	5.6	5.7	0.783	5.9	5.7	5.7	5.5	0.070	5.5	5.5	5.6	5.8	0.167
	Vinegar	5.7 ^a^	5.8 ^a^	6.2 ^b^	5.8 ^a^	**0.022**	5.8	5.5	5.9	5.9	0.059	5.8	6.1	5.7	5.8	0.121	5.8	5.6	5.8	5.9	0.194
Sweets	Black locust honey	6.7 ^a^	6.5 ^a^	7 ^b^	6.4 ^a^	**0.013**	6.7	6.7	6.7	6.6	0.939	6.7	6.4	6.6	6.7	0.386	6.6	6.7	6.5	6.7	0.650
	Chestnut honey	6.2	6.2	6.5	6	0.189	6.2	6.3	6.4	6.1	0.489	6.6	6.2	6.1	6.2	0.073	6.2	6.3	6.2	6.3	0.682
	Chocolate ice cream	6.9	6.8	7	6.7	0.298	6.9	6.9	6.7	6.8	0.544	7.2	6.9	6.9	6.9	0.183	6.9	6.8	6.8	7	0.392
	Dark chocolate	7.2	7.1	7.6	7.4	0.051	7.2	7.2	7.2	7.1	0.882	8.2 ^c^	7.6 ^b^	7.4 ^b^	7 ^a^	**<0.001**	7.1	7.3	7.2	7.4	0.214
	Dark chocolate pudding	6.3	6.4	6.4	6.2	0.832	6.3	6.4	6.3	6.2	0.826	7.2 ^c^	6.5 ^b^	6.4 ^b^	6.1 ^a^	**<0.001**	6.3	6.4	6.3	6.3	0.890
	Milk chocolate	6.9	6.7	7	6.6	0.136	6.8	6.9	7	6.9	0.479	6.2 ^a^	6.4 ^ab^	6.6 ^b^	7.1 ^c^	**<0.001**	6.9	6.7	6.7	6.7	0.126

## Data Availability

The data generated and/or analyzed during the current study are available from the corresponding author on reasonable request.

## Supplementary Materials

The following are available online at https://www.mdpi.com/2304-8158/11/1/5/s1, Figure S1: Differences between clusters (SNC = Strong Negative Correlation, WNC = Weak Negative Correlation, WPC = Weak Positive Correlation, and SPC = Strong Positive Correlation) in terms of the level of liking for the four samples are reported for each food model: Pear Juice (a), Bean Purée (b), Chocolate Pudding (c), and Tomato Juice (d). Within each sample, different letters indicate a statistically significant difference (*p* < 0.05) among clusters. Figure S2: Differences between clusters (SNC = Strong Negative Correlation, WNC = Weak Negative Correlation, WPC = Weak Positive Correlation, and SPC = Strong Positive Correlation) in terms of the perceived intensity of a target sensation among the four samples are reported for each food model: Pear Juice (a), Bean Purée (b), Chocolate Pudding (c), and Tomato Juice (d). Within each sample, different letters indicate a statistically significant difference (*p* < 0.05) among clusters. Table S1: List of abbreviations in alphabetical order.
