# Correction: Endrizzi et al. Relationships between Intensity and Liking for Chemosensory Stimuli in Food Models: A Large-Scale Consumer Segmentation. *Foods* 2022, *11*, 5

**DOI:** 10.3390/foods11152174

**Published:** 2022-07-22

**Authors:** Isabella Endrizzi, Danny Cliceri, Leonardo Menghi, Eugenio Aprea, Mathilde Charles, Erminio Monteleone, Caterina Dinnella, Sara Spinelli, Ella Pagliarini, Monica Laureati, Luisa Torri, Alessandra Bendini, Tullia Gallina Toschi, Fiorella Sinesio, Stefano Predieri, Flavia Gasperi

**Affiliations:** 1 Department of Food Quality and Nutrition, Research and Innovation Centre, Fondazione Edmund Mach, Via Edmund Mach 1, 38010 San Michele all’Adige, Italy; leonardo.menghi@fmach.it (L.M.); eugenio.aprea@fmach.it (E.A.); mathildeccharles@gmail.com (M.C.); flavia.gasperi@fmach.it (F.G.); 2 Center Agriculture Food Environment, University of Trento/Fondazione Edmund Mach, Via Edmund Mach 1, 38010 San Michele all’Adige, Italy; danny.cliceri@gmail.com; 3 Department of Technology and Innovation, Center University of Southern Denmark, Campusvej 55, 5230 Odense, Denmark; 4 Department of Agricultural, Food, Environment and Forestry (DAGRI), University of Florence, Via Donizetti 6, 50144 Florence, Italy; erminio.monteleone@unifi.it (E.M.); caterina.dinnella@unifi.it (C.D.); sara.spinelli@unifi.it (S.S.); 5 Department of Food, Environmental and Nutritional Sciences (DeFENS), University of Milan, 20133 Milan, Italy; ella.pagliarini@unimi.it (E.P.); monica.laureati@unimi.it (M.L.); 6 University of Gastronomic Sciences, Piazza Vittorio Emanuele II, 9, 12042 Pollenzo, Italy; l.torri@unisg.it; 7 Department of Agricultural and Food Sciences (DISTAL), Alma Mater Studiorum—University of Bologna, 40126 Bologna, Italy; alessandra.bendini@unibo.it (A.B.); tullia.gallinatoschi@unibo.it (T.G.T.); 8 CREA, Council for Agricultural Research and Economics, Research Center Food & Nutrition, Via Ardeatina 546, 00178 Rome, Italy; fiorella.sinesio@crea.gov.it; 9 Institute for Bioeconomy, CNR, National Research Council, Via Gobetti 101, 40129 Bologna, Italy; stefano.predieri@ibe.cnr.it

## Error in Figures

In the original publication [1], there was a mistake in “Figure 2, Figure 3, Figure 4, and Figure 5” as published. “In the mentioned figures, letters indicating the statistical significance of the differences in intensity/liking between the samples in each cluster are completely missed, and if reported they refer to the statistical significance between clusters instead of between samples”. The corrected “Figure 2, Figure 3, Figure 4, and Figure 5” appear below. The authors apologize for any inconvenience caused and state that the scientific conclusions are unaffected. This correction was approved by the Academic Editor. The original publication has also been updated.

**Figure 2:**
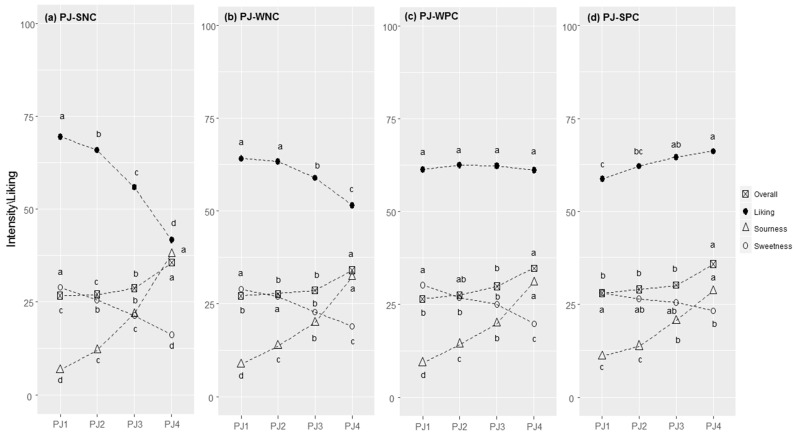
Pear juice (PJ) responses in each cluster (SNC = Strong Negative Correlation (**a**), WNC = Weak Negative Correlation (**b**), WPC = Weak Positive Correlation (**c**), and SPC = Strong Positive Correlation (**d**)): perceived intensity (gLM scale) and liking (LAM scale) averages for each concentration level of citric acid (1–4). Within each cluster, different letters indicate significant differences in intensity/liking between concentration levels (*p* < 0.05).

**Figure 3:**
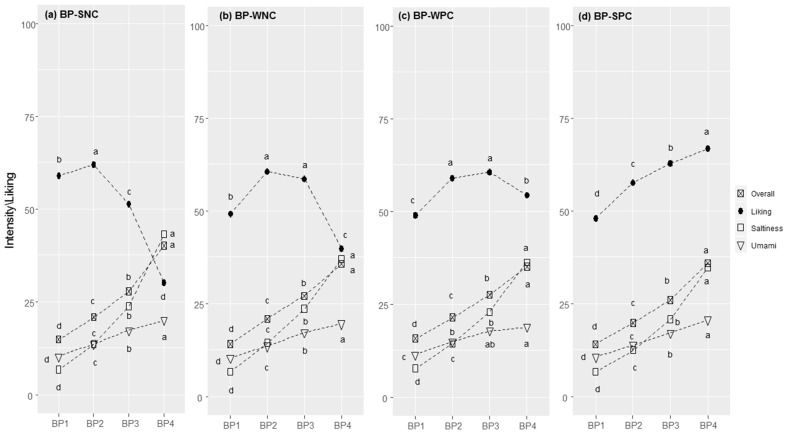
Bean purée (BP) responses in each cluster (SNC = Strong Negative Correlation (**a**), WNC = Weak Negative Correlation (**b**), WPC = Weak Positive Correlation (**c**), and SPC = Strong Positive Correlation (**d**)): perceived intensity (gLM scale) and liking (LAM scale) for bean purée samples at increasing concentrations of sodium chloride (1–4). Within each cluster, different letters indicate significant differences in intensity/liking between concentration levels (*p* < 0.05).

**Figure 4:**
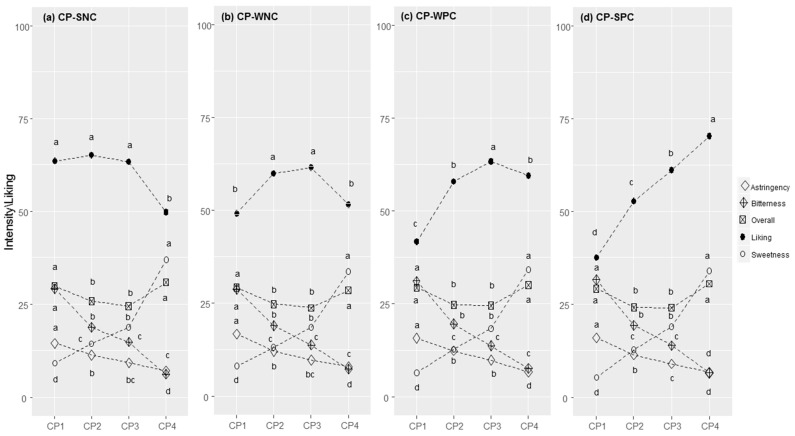
Chocolate Pudding (CP) responses in each cluster (SNC = Strong Negative Correlation (**a**), WNC = Weak Negative Correlation (**b**), WPC = Weak Positive Correlation (**c**), and SPC = Strong Positive Correlation (**d**)): perceived intensity (gLM scale) and liking (LAM scale) for samples at increasing concentrations of sucrose (1–4). Within each cluster, different letters indicate significant differences in intensity/liking between concentration levels (*p* < 0.05).

**Figure 5:**
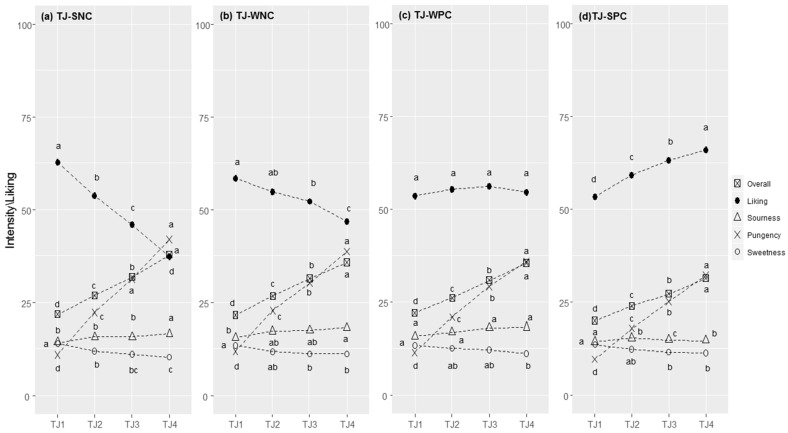
Tomato juice (TJ) responses in each cluster (SNC = Strong Negative Correlation (**a**), WNC = Weak Negative Correlation (**b**), WPC = Weak Positive Correlation (**c**), and SPC = Strong Positive Correlation (**d**)): perceived intensity (gLM scale) and liking (LAM scale) for samples at increasing concentrations of capsaicin (1–4). Within each cluster, different letters indicate significant differences in intensity/liking between concentration levels (*p* < 0.05).
