# Supplementary material for: Relationships between Intensity and Liking for Chemosensory Stimuli in Food Models: A Large-Scale Consumer Segmentation
Source: Foods. 2021 Dec 21;11(1):5. doi: 10.3390/foods11010005 (PMC8750454; doi:10.3390/foods11010005)
Supplement: Supplementary file 1 [file foods-11-00005-s001.zip › foods-1499489-supplementary.pdf]

foods

Supplementary materials

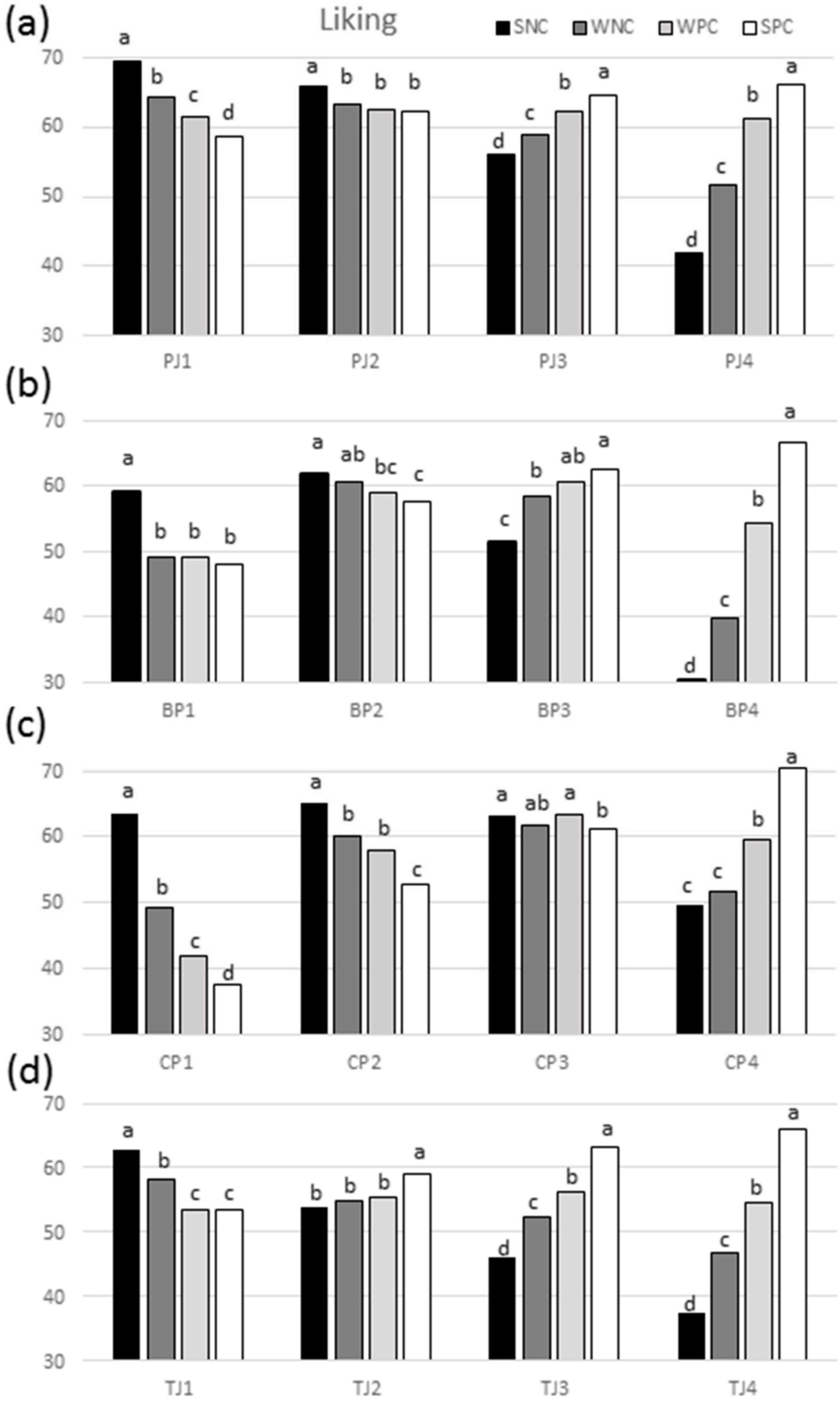

Figure S1. Differences between clusters (SNC = Strong Negative Correlation, WNC = Weak Negative Correlation, WPC = Weak Positive Correlation, and SPC = Strong Positive Correlation) in terms of liking among the four samples are reported for each food model: Pear Juice (a), Bean Purée (b), Chocolate Pudding (c), and Tomato Juice (d). Within each sample, different letters indicate a statistically significant difference (p<0.05) among clusters.

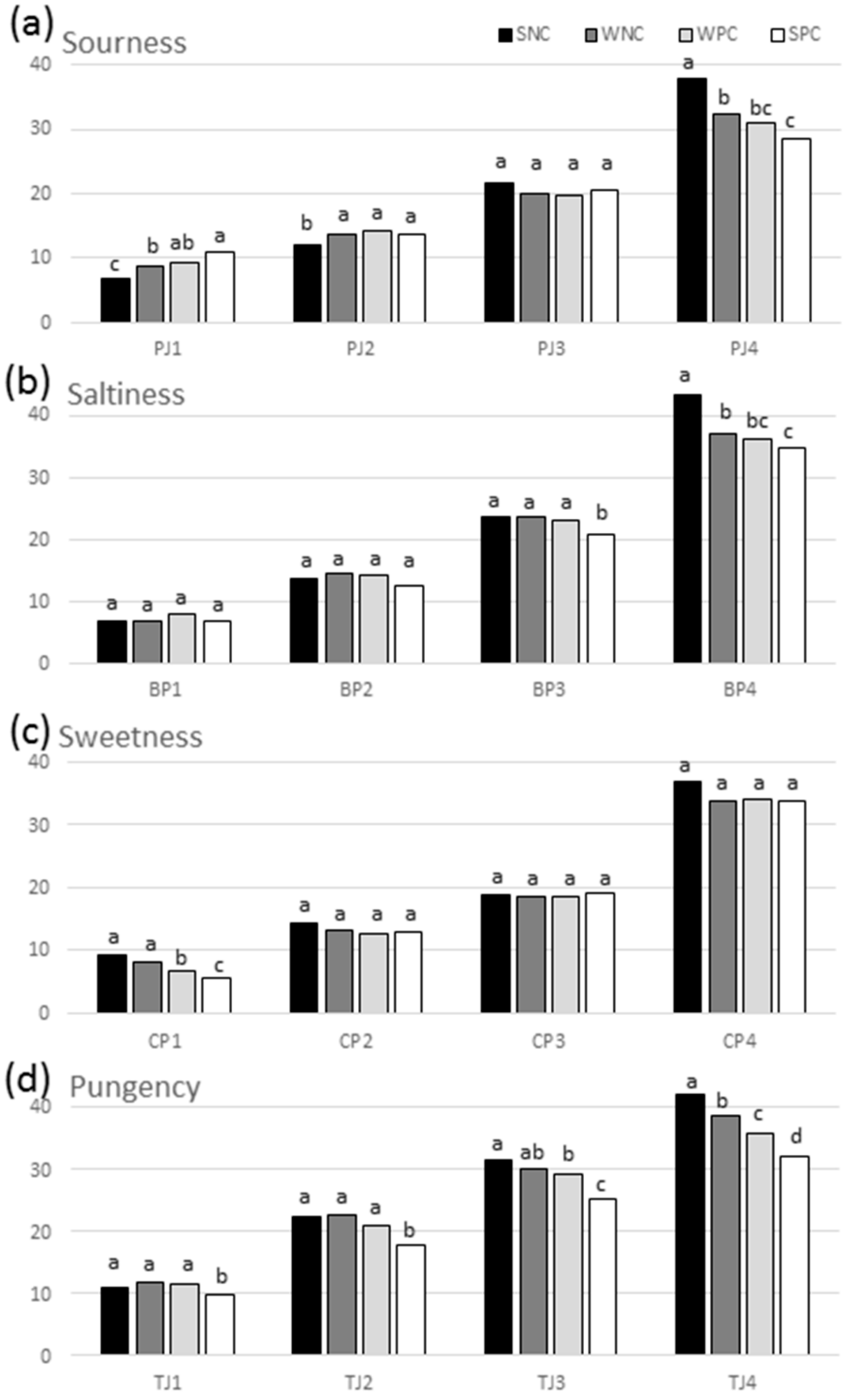

Figure S2. Differences between clusters (SNC = Strong Negative Correlation, WNC = Weak Negative Correlation, WPC = Weak Positive Correlation, and SPC = Strong Positive Correlation) in terms of perceived intensity of target sensation among the four samples are reported for each food model: Pear Juice (a), Bean Purée (b), Chocolate Pudding (c), and Tomato Juice (d). Within each sample, different letters indicate a statistically significant difference ($p<0.05$) among clusters.

Table S1. List of abbreviations in alphabetical order.

**Abbreviation**	**Description**
ANOVA	ANalysis Of VAriance
BMI	Body Mass Index
BP	Bean Purée
BP1- BP4	Bean purée samples with increasing concentration of sodium chloride
CP	Chocolate Pudding
CP1-CP4	Chocolate pudding samples with increasing concentration of sucrose
gLMS	general Labeled Magnitude Scale
IT	Italian Taste
LAM	Labelled Affective Magnitude
MT	medium tasters
NT	non-tasters
PJ	Pear Juice
PJ1-PJ4	Pear juice samples with increasing concentration of citric acid
PROP	6-n-propylthiouracil
SD	Standard Deviation
SNC	Strong Negative Correlation
SPC	Strong Positive Correlation
ST	supertasters
TJ	Tomato Juice
TJ1-TJ4	Tomato juice samples with increasing concentration of capsaicin
TS	Target sensation
WNC	Weak Negative Correlation
WPC	Weak Positive Correlation
